# Androgens as therapy for androgen receptor-positive castration-resistant prostate cancer

**DOI:** 10.1186/1423-0127-18-63

**Published:** 2011-08-23

**Authors:** Chih-Pin Chuu, John M Kokontis, Richard A Hiipakka, Junichi Fukuchi, Hui-Ping Lin, Ching-Yu Lin, Chiech Huo, Liang-Cheng Su

**Affiliations:** 1 Institute of Cellular and System Medicine, National Health Research Institutes, Miaoli, Taiwan; 2 Translational Center for Glandular Malignancies, National Health Research Institutes, Miaoli, Taiwan; 3 Ben May Department for Cancer Research, The University of Chicago, Chicago, USA; 4 Pharmaceuticals and Medical Devises Agency, Tokyo, Japan; 5 Department of Life Sciences, National Central University, Chungli, Taiwan

## Abstract

Prostate cancer is the most frequently diagnosed non-cutaneous tumor of men in Western countries. While surgery is often successful for organ-confined prostate cancer, androgen ablation therapy is the primary treatment for metastatic prostate cancer. However, this therapy is associated with several undesired side-effects, including increased risk of cardiovascular diseases. Shortening the period of androgen ablation therapy may benefit prostate cancer patients. Intermittent Androgen Deprivation therapy improves quality of life, reduces toxicity and medical costs, and delays disease progression in some patients. Cell culture and xenograft studies using androgen receptor (AR)-positive castration-resistant human prostate cancers cells (LNCaP, ARCaP, and PC-3 cells over-expressing AR) suggest that androgens may suppress the growth of AR-rich prostate cancer cells. Androgens cause growth inhibition and G1 cell cycle arrest in these cells by regulating c-Myc, Skp2, and p27^Kip ^via AR. Higher dosages of testosterone cause greater growth inhibition of relapsed tumors. Manipulating androgen/AR signaling may therefore be a potential therapy for AR-positive advanced prostate cancer.

## Introduction

In 1941, Huggins and Hodges reported that androgen ablation therapy causes regression of primary and metastatic prostate cancer [1]. Approximately 20-40% of patients treated with radical prostatectomy will have tumor recurrence and elevation of serum prostate-specific antigen (PSA) [2]. Primary metastatic sites for prostate cancer include bones and lymph nodes. More than 80% of patients who die from prostate cancer develop bone metastases [3-5]. Androgen ablation therapy is provided to patients who develop recurrent or metastatic prostate tumors. However, 80-90% of the patients who receive androgen ablation therapy ultimately develop recurrent castrate-resistant tumors 12-33 months after androgen ablation therapy. The median overall survival of patients after tumor relapse is 1-2 years [6,7]. Several long-term studies have failed to show that androgen ablation therapy provides a disease-specific survival advantage in patients [6]. Androgen ablation therapy is associated with undesired side-effects that impair the patient's quality of life as well as increased risk of diabetes and cardiovascular diseases [6]. Therefore, shortening the period of androgen ablation therapy may protect the patients.

### Androgens and Androgen Receptor in Prostate Cancer

Androgens are male sex hormone and include several steroids, such as testosterone, dehydroepiandrosterone, androstenedione, androstenediol, androsterone, and dihydrotestosterone (DHT). 90-95% of androgens are produced by the testes, while some androgens are produced in the adrenal glands. Testosterone is the main circulating androgen in human body, while DHT is a more potent androgen that has 5-fold higher affinity for the androgen receptor (AR) than does testosterone [7-9]. When testosterone enters prostate cells, 90% is converted to dihydrotestosterone (DHT) by the enzyme 5α-reductase [9].

The average serum testosterone level declines with age and elderly men usually have the condition as partial androgen deficiency. It decreases from approximately 620-670 ng/dl at age 25-44 to 470-520 ng/dl at age 65-84 [10]. A low serum testosterone level is associated with an increased risk of prostate cancer [11], and prostate tumors arising in a low testosterone environment appear to be more aggressive [12]. A retrospective review of 117 patients by Hoffman et al. revealed that patients with low (150 ng/dl) free testosterone have an increased percentage of biopsies with cancer present (43% versus 22%, p = 0.013) as well as an increased incidence of a biopsy with Gleason score of 8 or greater (7 of 64 versus 0 of 48, p = 0.025) [13]. These observations suggest that patients with prostate cancer and low free testosterone have more extensive disease, and low serum free testosterone may be a marker for more aggressive disease [13].

Androgen receptor (AR), an androgen-activated transcription factor, belongs to the nuclear receptor superfamily. Binding of DHT to the androgen receptor (AR) induces dissociation of AR from heat-shock proteins (HSPs) and stimulates AR phosphorylation [14]. AR dimerizes, translocates into the nucleus, and binds to androgen-response elements (ARE) in the promoter regions of target genes [14]. Co-activators and co-repressors also bind the AR complex, facilitating or preventing transcription of AR target genes. Activation or repression of target genes regulates growth, survival, and the production of prostate-specific antigen (PSA) in prostate cells [15,16].

Based on gene microarray studies of seven different human prostate cancer xenograft models, an increase of AR mRNA was the only change consistently associated with the development of the castration-resistant phenotype [17]. Increase in AR mRNA and protein is both necessary and sufficient to convert prostate cancer growth from a hormone-sensitive to a hormone-refractory stage, and is dependent on a functional ligand-binding domain [16,17]. Elevated AR expression in hormone-refractory prostate cancer cells or recurrent hormone-refractory tumors is observed in our progression model [15,18-22] and by several other groups [17,23-35]. Recent studies revealed that although androgen deprivation therapy significantly reduced serum testosterone concentrations, levels of testosterone and dihydrotestosterone occur in recurrent prostate cancer tissue are sufficient to stimulate AR transcription, PSA secretion, and tumor growth. These observations suggested that prostate cancer cells may survive androgen deprivation therapies by increasing intracrine androgen synthesis within the prostate [36,37].

### Androgen Ablation Therapy

Androgen ablation therapy, using luteinizing hormone-releasing hormone agonists (LH-RH) (also known as gonadotropin-releasing hormone, GnRH) or bilateral orchiectomy, has become a primary treatment for metastatic prostate cancer [6]. More than 80% of men with advanced prostate cancers respond to androgen ablation therapy, resulting in tumor shrinkage and reduction of serum PSA [6]. Anti-androgens are frequently used in conjunction with androgen ablation therapy as a combined androgen blockade to improve therapeutic outcome. Most patients experience an initial rapid decline in PSA followed by a slower decline to the nadir. The initial rapid decrease in PSA results from the cessation of androgen-regulated PSA synthesis and apoptosis of prostate cancer cells, while the ongoing slower decline perhaps reflects decreasing tumor volume [38]. Anti-androgen finasteride prevents and delays the appearance of prostate cancer observed in a prevention trial with 18,882 men, however, tumors of higher Gleason grade (7-10) were more common in the finasteride group (37%) than in the placebo control group (22%) [39].

In addition, androgen deprivation therapy is associated with several undesired side-effects, including sexual dysfunction, osteoporosis and bone fractures, hot flashes, fatigue, gynecomastia, anemia, depression, cognitive dysfunction, increased risk of diabetes, and cardiovascular diseases [6,40-42]. Androgen deprivation therapy using LH-RH agonists increases risk of incident diabetes, incident coronary heart disease, myocardial infarction, sudden cardiac death, and stroke [43-45]. Combined androgen blockade (LH-RH agonists treatment plus oral anti-androgens) is associated with increased risk of incident coronary heart disease [42]. Orchiectomy is associated with coronary heart disease and myocardial infarction [42]. Therefore, shortening the period of androgen ablation therapy may be beneficial for some prostate cancer patients.

### Intermittent Androgen Deprivation Therapy

Clinical and basic studies have shown that in comparison with continuous androgen ablation (CAB) therapy, Intermittent Androgen Deprivation (IAD) therapy substantially prolongs the time to development of castration-resistant prostate cancer [39,46-48]. Intermittent Androgen Deprivation therapy is a strategy to periodically perform and terminate the androgen ablation therapy, therefore patients in "off-androgen ablation therapy" periods may decrease undesired side effects and improve quality of life.

The growth of Shionogi mammary carcinoma is stimulated by androgens and was the first experimental model to test IAD therapy. Hormone-dependent Shionogi mammary carcinoma become androgen ablation-resistant following IAD therapy using cycles of transplantation into intact male mice followed by castration [49]. However, IAD delayed the recurrence time of Shionogi tumor growth from 51 days to 147 days [46]. Five to six cycles of IAD therapy delays the progression of LNCaP prostate xenografts towards androgen ablation-resistance. IAD prolongs the time to androgen ablation-resistance of PSA gene regulation from an average of 26 days to 77 days compared to continuous androgen ablation (CAB) [47]. By 15 weeks post-castration, serum PSA levels increase 7-fold above pre-castrate levels in CAB-treated mice compared to a 1.9-fold increase in IAD-treated mice [47].

In a Canadian Prospective Trial, Bruchovsky et al. showed that IAD therapy causes repeated differentiation of prostate tumors with recovery of apoptotic potential, inhibition of tumor growth after rapid restoration of serum testosterone, and restraint of tumor growth by subnormal levels of serum testosterone [43]. Pether et al. reported in a clinical trial of 102 patients that there is a trend toward extended times to progression and death compared to CAB treatment, and growth of advanced prostate tumors is delayed in ~50% patients treated with IAD [45]. They concluded that IAD is a viable treatment option for men with prostate cancer which affords an improved quality of life when the patient is off therapy and with reduced toxicity and costs [43-45].

### Androgenic Suppression of Advanced Prostate Cancer Cells *in Vitro*

The delay of progression toward androgen-independency in IAD treatment might be related to the suppressive effect of androgen on AR-positive hormone-refractory prostate cancer cells that is observed in the LNCaP and other prostate cancer cell models. LNCaP is one of the most commonly used cell lines for prostate cancer research and was derived from a human lymph node metastatic lesion of prostate adenocarcinoma [49,50]. LNCaP cells express AR and PSA. To establish relapsed androgen-ablation resistant prostate cancer cells that mimic the clinical situation in which prostate cancer recurs during androgen deprivation, we cultured androgen-sensitive LNCaP 104-S cells in androgen-depleted conditions *in vitro *[19,20]. After 20 passages (3 months) in androgen-depleted media supplemented with dextran-coated charcoal-stripped fetal bovine serum, most LNCaP 104-S cells undergo cell cycle arrest. After 60-80 passages (8-11 months), cells called 104-R1 cells emerge that grow much more rapidly in the absence of androgen. After 120-150 passages (16-20 months) in androgen-depleted medium, 104-R1 cells give rise to cells called 104-R2 cells, that proliferate in the absence of androgen at a rate comparable to the proliferation rate of 104-S cells grown in media with androgen [19,20].

During the transition of 104-S cells to 104-R1 and 104-R2 cells, AR mRNA and protein levels increase. AR transcriptional activity also increases several fold [15,18-20,51]. Proliferation of 104-R1 and 104-R2 cells is not dependent on androgen (i.e. hormone-refractory) but is unexpectedly suppressed by physiological concentrations of androgen both *in vitro *and *in vivo *[15,18-22,51]. When 104-R1 or 104-R2 cells are incubated for several weeks in a high concentration of R1881 (20 nM, approximately equivalent to 200 nM DHT), cells adapt after a period of growth arrest to grow at a rate equivalent to the parental 104-R1 or 104-R2 cells [20,51]. The adapted cells derived from 104-R1 called R1Ad cells, which grow optimally in 10 nM R1881 [26]. R2Ad cells, which derived from 104-R2 cells under androgen treatment, grow androgen-insensitively [51]. R1Ad and R2Ad cells have dramatically reduced levels of AR, which suggests that elevated AR expression is responsible for the repressive effect of androgen in 104-R1 and 104-R2 cells.

To further mimic the clinical situation of combined androgen deprivation and anti-androgen therapy, LNCaP 104-S cells were incubated with 5 μM Casodex in androgen-depleted medium. After four weeks, Casodex-resistant colonies (CDXR cells) appear at low frequency (1 in 1.4 × 10^5^) as most of the cells appear to undergo senescent cell death [21]. Like 104-R1 and 104-R2 cells, CDXR cells have increased AR expression and activity and are repressed by androgen [21]. Unlike 104-R1 cells, CDXR cells grown in 10 nM R1881 undergo apoptotic cell death starting 6 to 8 days after R1881 exposure. However, 1 in 1.9 × 10^3 ^cells form colonies of androgen-insensitive cells that are not repressed by R1881 or Casodex. These sublines, designated IS cells, show greatly reduced AR expression [27]. Unlike R1Ad cells, the growth of IS cells is not stimulated by R1881. IS cells are more similar to R2Ad cells. During progression from 104-R1 to 104-R2 stages, the cells appear to pass a point where cells can no longer recover responsiveness to androgen, but instead progress to androgen insensitivity [52]. Direct progression of 104-S cells to the CDXR stage by selection in anti-androgen seems to bypass this intermediate 104-R1 stage and speed up the diseases progression. Stimulation of prostate cancer disease progression by antiandrogen treatment is also observed in clinical trials. Bales et al. compared the effect of bicalutamide (50 mg daily) to surgical or medical castration in three randomized trials involving more than 1000 patients and found that treatment with bicalutamide resulted in a statistically significant shorter time to treatment failure, time to progression, and median survival compared to castration (hazard ratios 1.59, 1.62, and 1.44, respectively) [53].

An androgen-suppressive phenotype of hormone-refractory LNCaP cells has been observed by several other groups [20,38,54-56]. Elevated AR is observed in hormone-refractory LNCaP cells [32,57,58]. In one study, the most optimal concentration of androgen for proliferation of cells at intermediate stage shifts from 0.01 nM R1881 to 0.001 nM R1881 [57]. The proliferation of the late stage hormone-refractory LNCaP cells is suppressed by androgen [57].

LNCaP cells express a mutant AR (T877A) that displays relaxed ligand binding specificity [20,59]. However, androgenic suppression is not limited to LNCaP cells. ARCaP is an AR-positive, tumorigenic, and highly metastatic cell line derived from the ascites fluid of a patient with advanced metastatic disease. Proliferation of ARCaP cells is suppressed by androgen [60]. ARCaP cells engineered to overexpress AR have a biphasic androgenic response, the cells are stimulated by low concentration of androgen (0.1-10 nM R1881), but suppressed by high concentration of androgen (100-1000 nM R1881) [61]. MDA PCa 2b-hr cells were generated *in vitro *from bone metastasis-derived, hormone-dependent MDA PCa 2b human prostate cancer cells after 35 weeks of culture in androgen-depleted medium. MDA PCa 2b-hr express 3-fold higher AR protein and proliferation of MDA PCa 2b-hr is stimulated by 3.5 nM testosterone or physiological concentrations of adrenal androgens but is inhibited by higher concentrations of testosterone or bicalutamide [31]. PC-3 is a commonly used AR-negative human prostate cancer cell line established from a bone-derived metastasis [50]. Physiological concentrations of DHT cause growth inhibition, G1 cell cycle arrest, and apoptosis in PC-3 cells overexpressing full length wild-type AR [62-64]. Much evidence therefore exists for AR functioning as a ligand-dependent tumor suppressor in prostate cancer cells when it is expressed at high levels and is fully activated.

### Androgenic Suppression of Advanced Prostate Cancer Cells *in Vivo*

**Figure 1:**
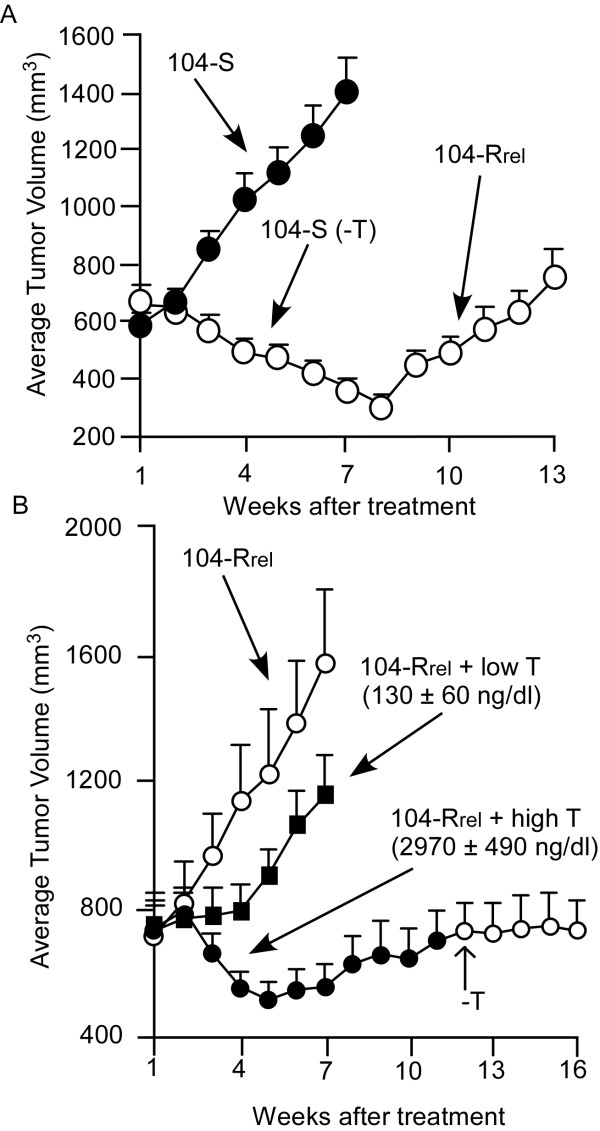
**Progression of hormone-dependent LNCaP 104-S tumors to androgen-ablation-resistant 104-Rrel tumors, and androgenic growth suppression of 104-Rrel tumors**. (A) Mice were injected subcutaneously with hormone-dependent 104-S cells. After allowing tumors to grow for 7 weeks, mice were separated into control (filled circles, 14 mice with 19 tumors) and castration groups (open circles, 24 mice with 36 tumors) and the time was designated as week 1 [18]. (B) Mice in the castrated group in (A) at the 14^th ^week were separated into 3 groups including a control group (open circles, 6 mice with 9 tumors), a low dosage testosterone treatment group that received a subcutaneous implant of a 20 mg Testosterone/cholesterol (1:9) pellet (filled squares, 9 mice with 12 tumors), and a high-dosage testosterone treatment group that received a subcutaneous implant of a 20 mg pure Testosterone pellet (filled circles, 10 mice with 12 tumors) [18]. Tumor volumes are expressed as the mean + standard error.

**Figure 2:**
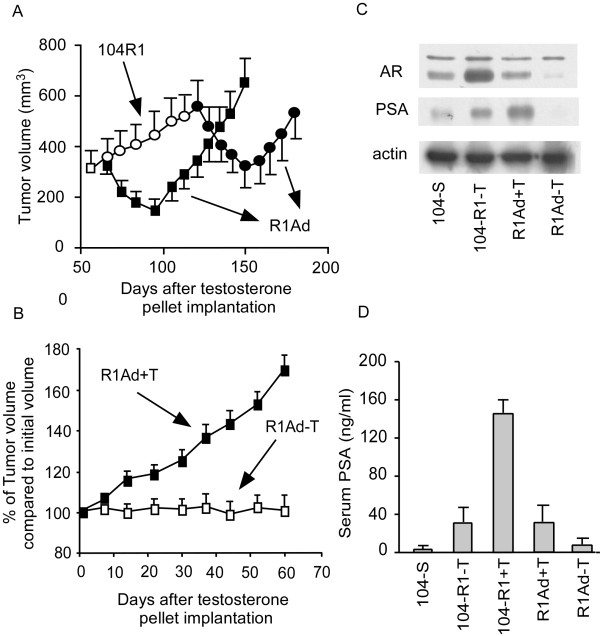
**Progression and regression of LNCaP 104-R1 tumor xenografts in nude mice treated with testosterone**. (A) LNCaP 104-R1 tumor xenografts in castrated male nude mice were allowed to grow until they reached an average volume of 300 mm^3 ^on the 58th day. On the 67th day, mice were separated into a control group (open circles) and a treatment group (filled circles). The treatment group received a subcutaneous implant of a 20 mg testosterone pellet. The mice in the control group were implanted with a 20 mg testosterone pellet on day 121. Open circles represent tumor in mice without testosterone, while filled circles and filled squares represent tumors in mice with testosterone. Tumor volumes are expressed as the mean ± standard error [15]. (B) For mice carrying adapted R1Ad tumors from (A), testosterone pellets were removed from 5 mice (10 tumors). Their tumor growth was compared with tumors in mice bearing testosterone pellets (5 mice with 10 tumors) [15]. (C) PSA, AR, and actin protein levels in 104-S tumor (in intact mice), 104-R1-T tumors, R1Ad-1+T tumors, and R1Ad-T were assayed by Western blot [15]. (D) Serum PSA level of mice with 104-S tumors (in intact mice), 104-R1-T tumors, 104-R1+T tumors, R1Ad+T tumors, R1Ad-T tumors was determined by ELISA [15].

**Figure 3:**
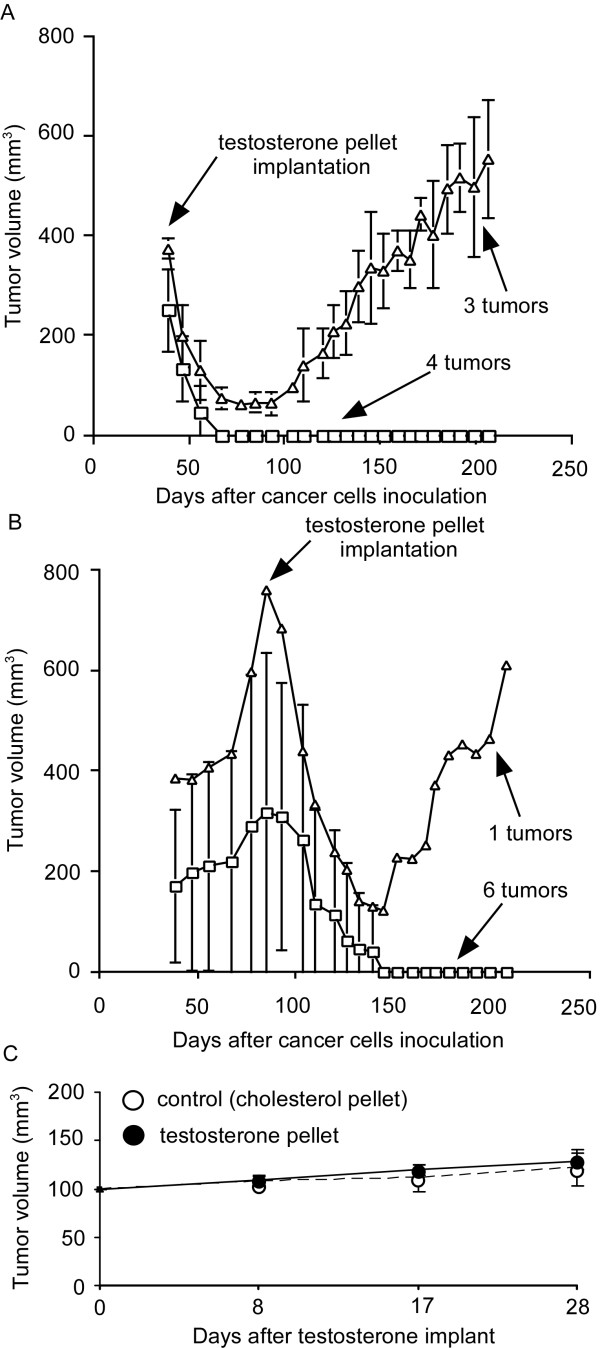
**Regression and relapse of LNCaP CDXR-3 tumor xenografts in nude mice treated with testosterone LNCaP CDXR tumor xenografts in castrated male nude mice were allowed to grow until they reached an average volume of 400 mm^3 ^on the 38th day**. All mice carrying tumors received a subcutaneous implant of a 20 mg testosterone pellet. The mice in the control group were implanted with a 20 mg testosterone pellet either at an early stage (50 days after inoculation, 7 tumors) (A) or late stage (92 days after inoculation, 7 tumors) (B) [27]. Open triangles represent tumors relapsed, while open squares represent tumors disappeared after androgen treatment. Tumor volumes are expressed as the mean ± standard error. (C) LNCaP IS-3 xenogarfts were separated into control group (20 mg cholesterol pellet implant, 9 tumors) and treatment group (20 mg testosterone pellet implant, 10 tumors) to determine the effect of androgen on growth of IS tumors [21].

Castration causes regression of 104-S xenografts, but tumors begin to regrow after 8 weeks as androgen ablation-resistant relapsed tumors called 104-Rrel with elevated AR mRNA and protein expression [18]. Low serum levels of testosterone (130 ± 60 ng/dl) stop growth of 104-Rrel tumors but tumor growth resumes in about 4 weeks. High serum levels of testosterone (2970 ± 495 ng/dl), which is approximately 5-fold higher than normal levels, cause regression of 104-Rrel tumors. However, 104-Rrel cells adapt to androgen and relapse after 4 weeks as androgen-stimulated 104-Radp tumors [18] (Figure 1). Growth of the LNCaP 104-R1 tumors is also suppressed by androgen, but tumors adapt to androgenic suppression and relapse as androgen-stimulated R1Ad tumors in 5-6 weeks [15] (Figure 2A, B). Growth of these tumors is stimulated by testosterone and removal of testosterone totally stopped the tumor growth [15,18]. Both 104-Radp and R1Ad tumors express very little AR and PSA mRNA and protein or serum PSA level (Figure 2C, D), similar to R1Ad cells in cell culture [15,18,20]. Xenograft of CDXR cells, which are also derived from 104-S cells, behave differently under androgen suppression compared to 104-R1 xenografts. Both early and late treatment with androgen causes regression of CDXR tumors. Approximately 70% of tumors regress completely and the rest of the tumors relapse after 60-90 days of treatment [27]. The relapsed tumors show diminished expression of AR and no longer require androgen for growth, essentially identical to the behavior of IS3 cells that emerged after androgen exposure *in vitro *[21]. It is worthwhile noting that 100% of 104-R1 tumor treated with testosterone relapse in 4-5 weeks, while only 30% of CDXR tumors and 70% of R2Ad tumors relapse after 9-13 and 4-5 weeks, respectively, after testosterone treatment [15,21,51] (Figure 3). This is probably due to the slower proliferation rate of CDXR cells and the apoptosis induced in CDXR cells but not 104-R1 cells by androgen [20,21]. Regression and relapse after androgen treatment of LNCaP xenograft is also observed by another group [64] and ARCaP xenograft [65]. AR overexpression decreases adhesion, invasion, and migration ability of ARCaP cells *in vitro*, as well as reduces ARCaP tumor growth in athymic mice [61].

### Molecular Mechanism of Androgenic Suppression

The anti-androgen Casodex, unlike flutamide and cyproterone acetate, does not exhibit agonist activity and acts as a true antiandrogen in the LNCaP 104-S, 104-R1, 104-R2 cell lines [66,67]. Casodex does not affect proliferation of 104-R1 and 104-R2 cells but blocks androgenic repression of growth as well as androgenic induction of PSA [68], suggesting that the growth inhibition caused by androgen treatment is via AR. Knockdown of AR expression in CDXR3 cells by shRNA, either constitutive or conditional, relieves androgenic repression of growth and does not affect cell growth in the absence of androgen [21]. Retroviral overexpression of AR in IS2 and IS3 cells, on the other hand, restores the androgen-repressed phenotype in these cells [21]. R2Ad cells show similar behavior compared to CDXR cells [51]. Conditional overexpression of AR in 104-S cells causes androgen-induced growth repression and does not confer hormone-refractory growth [21]. These observations confirm that androgen causes growth inhibition via AR.

**Figure 4:**
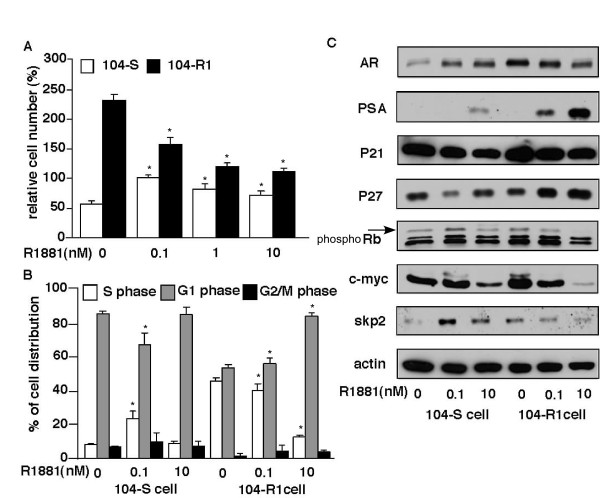
**Effect of androgen on cell proliferation, cell cycle, and cell cycle-related proteins in hormone-dependent 104-S and androgen ablation-resistant 104-R1 cells**. (A) LNCaP 104-S and 104-R2 cells were treated with increasing concentration of R1881 for 96 hours. Relative cell number was determined by using a 96-well proliferation assay and data were normalized to number of 104-S cells at 0.1 nM R1881. Asterisk (*) represents statistically significant difference between treatment group compared to control group of 104-S or 104-R1 cells. (B) Percentage of 104-S and 104-R1 cells in S phase determined by flow cytometry. LNCaP 104-S and 104-R2 cells were treated with increasing concentrations of R1881 for 96 hours. Values represent the mean + standard error derived from 5 independent experiments. (C) Protein expression of androgen receptor (AR), prostate specific antigen (PSA), p21^cip^, p27^Kip^, phosphor-retinoblastoma protein (Rb), c-Myc, S phase kinase-associated protein 2 (Skp2) were determined by Western blotting assay in 104-S and 104-R1 cells treated 96 hrs with different concentration of R1881. β-actin was used as loading control.

Flow cytometric analysis of androgen-treated cells reveals that androgen treatment of hormone-dependent LNCaP FGC [54] or LNCaP 104-S cells [20] relieves a G1 arrest induced by androgen deprivation. Conversely, R1881 induces G1 arrest in 104-R1 and 104-R2 cells beginning after about 24 hours of exposure [20] (Figure 4) as well as other LNCaP model [55,58]. Casodex blocks the effect of androgen in all cell lines. Expression of known cdk inhibitors (p15, p16, p18, p19, and p21^waf1/cip1^, p27^Kip1^, p57^Kip2^) has been examined in 104-S, 104-R1, and 104-R2 cells treated with or deprived of androgen. p21^waf1/cip1 ^and p27^Kip1 ^levels are induced by androgen in 104-R1 and 104-R2 cells [20,51] (Figure 4). p21^waf1/cip1 ^is induced transiently in 104-R1 cells only, while p27^Kip1 ^is induced persistently about 3-fold in both 104-R1 and 104-R2 cells [20,51]. Similar results have been obtained with the CDXR sublines [27]. In contrast, expression of p21^waf1/cip1 ^and p27^Kip1 ^is repressed by androgen in 104-S cells. Androgens regulate expression of the F-box protein Skp2 that binds phosphorylated p27^Kip1 ^[59,60,69] leading to its ubiquitination and proteolysis. Androgen down-regulates Skp2 in 104-R1, 104-R2 (Figure 4) [51] and CDXR cells, which leads to accumulation of p27^Kip1^. Androgen treatment down-regulates c-Myc mRNA and protein expression in hours in 104-R1 and 104-R2 cells (Figure 4)[51], and enforced retroviral overexpression of Skp2 or c-Myc blocks androgenic repression of 104-R1 growth [19,51]. c-Myc may have an indirect effect on p27^Kip1 ^expression through the induction of Cks1, a component of the SCF^Skp2 ^complex responsible for p27 ^Kip1 ^degradation [70]. Therefore, androgen regulates cell cycle and proliferation of LNCaP cells via AR, Skp2, c-Myc, and p27^Kip1^.

### Androgen Treatment of Prostate Cancer

Reduced serum testosterone levels by androgen ablation therapy causes regression of prostate tumors, but elevation of the testosterone level does not result in stimulation of tumor growth or secretion of PSA [71]. A few studies have shown that androgen is safe and potentially effective for treatment of advanced prostate cancer. Mathew reported that the testosterone level in a prostate cancer patient that had undergone radical prostatectomy and LH-RH therapy remained at castrated levels and serum PSA was undetectable for 15 years. PSA levels then began to rise and the patient was given testosterone replacement therapy to attain a normal range of serum testosterone. After an initial flare, PSA levels gradually declined over 18 months. After 27 months, PSA level started to increase. When testosterone replacement therapy was discontinued, PSA levels dropped [48]. Mathew agrees that the observation was somewhat similar to the transition from 104-R1 to R1Ad phenotype under androgen treatment in our LNCaP progression model [15,20,48].

Szmulewitz et al. randomly separated 15 prostate cancer patients (median PSA of 11.1 ng/ml, range from 5.2-63.6 ng/ml) who received androgen ablation plus anti-androgen therapy and withdrew without metastatic disease into three groups. The three groups of patients were given treatment of three different dosages of transdermal testosterone: 2.5, 5.0, or 7.5 mg/day. Testosterone increased from castration levels to median concentrations of 305 ng/dl, 308 ng/dl, and 297 ng/dl for dosages of 2.5 mg/day (n = 4), 5.0 mg/day (n = 5), and 7.5 mg/day (n = 5), respectively. One patient was taken off due to grade 4 cardiac toxicity. One patient experienced symptomatic progression, and three (20%) patients demonstrated a decrease in PSA (largest was 43%). Median time to progression was 9 weeks (range: 2-96), with no detectable difference in the three dose cohorts [39]. The conclusion of this study is that testosterone is a feasible and reasonably well-tolerated therapy for men with early hormone-refractory prostate cancer [39]. Aromatase inhibitors were not applied to prevent the conversion of testosterone to estradiol (E2) by aromatase, and elevation of estradiol may be responsible for the cardiac toxicity [72].

A phase 1 clinical trial was performed to determine the safety of high-dose exogenous testosterone in patients with castration-resistant metastatic prostate cancer. Patients with progressive castration-resistant prostate cancer who had been castrated for at least 1 yr received three times the standard replacement dose of transdermal testosterone by skin patch or topical gel. No adverse effects were reported. Cohorts of 3-6 patients received testosterone for 1 week, 1 month, or until disease progression. Average testosterone levels were within normal physiological concentration. The serum testosterone ranged from 330-870 ng/dl. One patient achieved a PSA decline of > 50% from baseline, although no other significant effect was observed. No difference was observed between different cohorts [73]. This study suggests that patients with advanced prostate cancer can be safely treated with exogenous testosterone. As patients on average did not achieve sustained supraphysiological serum testosterone levels, future studies maximizing testosterone serum levels in selected patients with AR overexpression may improve the treatment outcome.

## Conclusions

**Figure 5:**
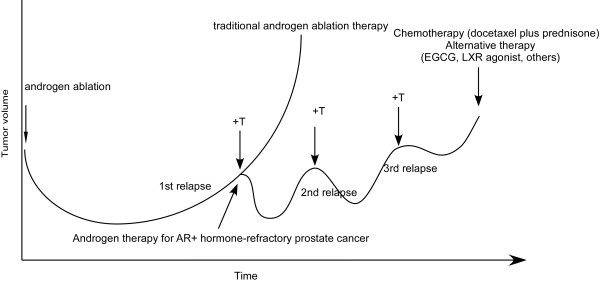
**Androgen and alternative therapy for advanced prostate cancer**. After androgen ablation therapy, androgen treatment will retard the growth and progression of AR-rich advanced tumors in patients. In that case, chemotherapy (docetaxel plus prednisone) or alternative therapies, such as EGCG, LXR agonist or other treatments, should be considered to suppress tumor growth.

Although our observations suggested that androgen suppress growth of AR-positive advanced prostate tumors while Vancouver group use IAD to show that cessation of anti-androgen therapy allowed tumor cells to recover their androgen-sensitivity and be sensitive to subsequent rounds of anti-ablation treatment. We believe that our LNCaP progression model may provide the molecular explanation for IAD treatment. As most prostate tumors relapsed from androgen ablation therapy express AR and expression of mRNA and protein level of AR are frequently elevated [23-25], restoration of endogenous testosterone level by IAD treatment will suppress the proliferation of AR-rich relapsed prostate cancer cells based on observations in LNCaP 104-R1, 104-R2, CDXR, and in other relapsed prostate cancer cell models [15,18-22,31,32,55,57,58,61-65,74]. The decrease in testosterone production is generally reversible upon cessation of LH-RH agonist therapy, however, testosterone production does not always return to baseline levels and may be related to the duration of LH-RH agonist therapy, patient age, and other factors [75,76]. According to our study, serum testosterone level around 2970 ± 495 ng/dl is required to cause regression of relapsed tumors [18], so patients showing no response to IAD treatment might be either having tumors expressing very low AR expression or having very low serum testosterone level. For the later ones, exogenous testosterone should be applied to patients to suppress the growth of relapsed tumors. At the beginning of IAD or testosterone treatment, serum PSA level will increase dramatically [48], similar to the stimulated PSA expression in 104-R1, 104-R2, and CDXR cells [15,18,20,21,51]. The AR-rich relapsed prostate cancer cells will then undergo G1 cell cycle arrest and/or apoptosis [25-27,59,64,65], causing the regression of tumor and decrease of serum PSA level [15,18,21,22]. The regression of tumors can continue for weeks or months before the prostate cancer cells adapt to the androgenic suppression [15,18,21,51,58], possibly by down-regulating AR [15,18,21,51]. The adapted cells are probably similar to R1Ad cells [15,18,20] in patients receiving androgen ablation therapy (LH-RH agonists) or similar to IS or R2Ad cells [21] in patients receiving combined treatment of LH-RH agonists and anti-androgens or long-term androgen ablation therapy. The stimulation of PSA secretion by androgen in R1Ad, R2Ad, or IS cells is very low, so the serum PSA level will remain low until the adapted tumors start to grow, either stimulated by testosterone like R1Ad cells or by androgen-insensitive growth like R2Ad and IS cells. IAD will delay the growth of R1Ad-like tumors [15,18,20] but not R2Ad or IS-like tumors [27]. Therefore, only the subgroup of patients carrying R1Ad-like tumors will respond to the subsequent cycles of IAD treatment. As 104-R1 cells will progress to 104-R2 cells in androgen-depleted medium and 104-R2 cells will progress to R2Ad cells following androgen treatment, patients receiving a few cycle of IAD treatment will ultimately develop androgen-insensitive tumors that will not respond to further IAD treatment [43-45,47]. Alternative therapies, such as chemotherapy (docetaxel plus prednisone) [77], green tea catechin epigallocatechin 3-gallate (EGCG), or liver X receptor agonists, might be able to suppress growth of these androgen-insensitive prostate tumors [18,50,78-82] (Figure 5).

Based on the results from our *in vitro *and *in vivo *progression model, patients developing relapsed hormone-refractory prostate tumors after androgen ablation therapy should be biopsied for expression level of AR protein in tumors. IAD and/or administration of exogenous androgen at a concentration 2500-3500 ng/dl will benefit patients with AR-rich relapsed tumors by suppressing tumor growth, improving quality of life, and reducing risks for cardiovascular diseases and diabetes. Combined treatment of androgen ablation therapy with anti-androgen cause a rapid and irreversible selection of more aggressive advanced prostate cancer cells [83], possibly similar to CDXR cells. Exogenous androgen treatment can cause regression of these tumors and a subgroup of these tumors will disappear [21]. Androgen deprivation therapy alone may promote a slow adaptation to androgen ablation-resistance [15,20], thus shortening the period of androgen deprivation therapy may retard the diseases progression and reduce side effects. Aromatase inhibitors should be considered in combination with androgen treatment to prevent the conversion of testosterone to estradiol (E2) by aromatase to avoid potential cardiac toxicity. Since several clinical trials already confirmed that testosterone is a safe, feasible, and reasonably well-tolerated therapy for men with early hormone-refractory prostate cancer [39,48,72,73], we believe that manipulating androgen/AR signaling can be a potential therapy for AR-positive advanced prostate cancer.

## Disclosure of Competing interests

The authors declare that they have no competing interests.

## Authors' contributions

All authors contributed to the writing, read, and approved the final manuscript.

## Endnotes

This article is dedicated to our dear mentor Dr. Shutsung Liao, professor at Ben May Department for Cancer Research of The University of Chicago for his 80^th ^birthday. He is a member of America Academy of Art & Science (U.S.A.) and academician of Academia Sinica (Taiwan).
